# Apparent Diffusion Coefficient-Based Convolutional Neural Network Model Can Be Better Than Sole Diffusion-Weighted Magnetic Resonance Imaging to Improve the Differentiation of Invasive Breast Cancer From Breast Ductal Carcinoma *In Situ*

**DOI:** 10.3389/fonc.2021.805911

**Published:** 2022-01-14

**Authors:** Haolin Yin, Yu Jiang, Zihan Xu, Wenjun Huang, Tianwu Chen, Guangwu Lin

**Affiliations:** ^1^ Department of Radiology, Huadong Hospital Affiliated to Fudan University, Shanghai, China; ^2^ Department of Radiology, West China Hospital, Sichuan University, Chengdu, China; ^3^ Lung Cancer Center, Cancer Center and State Key Laboratory of Biotherapy, West China Hospital of Sichuan University, Chengdu, China; ^4^ Department of Radiology, Affiliated Hospital of North Sichuan Medical College, Nanchong, China

**Keywords:** breast cancer, ductal carcinoma *in situ*, diffusion-weighted imaging, magnetic resonance imaging, deep learning

## Abstract

**Background and Purpose:**

Breast ductal carcinoma *in situ* (DCIS) has no metastatic potential, and has better clinical outcomes compared with invasive breast cancer (IBC). Convolutional neural networks (CNNs) can adaptively extract features and may achieve higher efficiency in apparent diffusion coefficient (ADC)-based tumor invasion assessment. This study aimed to determine the feasibility of constructing an ADC-based CNN model to discriminate DCIS from IBC.

**Methods:**

The study retrospectively enrolled 700 patients with primary breast cancer between March 2006 and June 2019 from our hospital, and randomly selected 560 patients as the training and validation sets (ratio of 3 to 1), and 140 patients as the internal test set. An independent external test set of 102 patients during July 2019 and May 2021 from a different scanner of our hospital was selected as the primary cohort using the same criteria. In each set, the status of tumor invasion was confirmed by pathologic examination. The CNN model was constructed to discriminate DCIS from IBC using the training and validation sets. The CNN model was evaluated using the internal and external tests, and compared with the discriminating performance using the mean ADC. The area under the curve (AUC), sensitivity, specificity, and accuracy were calculated to evaluate the performance of the previous model.

**Results:**

The AUCs of the ADC-based CNN model using the internal and external test sets were larger than those of the mean ADC (AUC: 0.977 vs. 0.866, P = 0.001; and 0.926 vs. 0.845, P = 0.096, respectively). Regarding the internal test set and external test set, the ADC-based CNN model yielded sensitivities of 0.893 and 0.873, specificities of 0.929 and 0.894, and accuracies of 0.907 and 0.902, respectively. Regarding the two test sets, the mean ADC showed sensitivities of 0.845 and 0.818, specificities of 0.821 and 0.829, and accuracies of 0.836 and 0.824, respectively. Using the ADC-based CNN model, the prediction only takes approximately one second for a single lesion.

**Conclusion:**

The ADC-based CNN model can improve the differentiation of IBC from DCIS with higher accuracy and less time.

## Introduction

Breast cancer is the most common malignant tumor in women worldwide and has the highest mortality rate among all malignant tumors in women (1). Breast ductal carcinoma *in situ* (DCIS) is the proliferation of malignant epithelial cells in ducts without involving the basement membrane (2). DCIS has no metastatic potential and has better clinical outcomes compared with invasive breast cancer (IBC) (3). Mammographic screening programs in many countries have led to a substantial increase in the early detection of DCIS, which accounts for 20–30% of newly detected breast cancers (4, 5). Higher detection rates have triggered anxiety concerning the problem of overdiagnosis and subsequent overtreatment. Therefore, the feasibility of pharmacological intervention may be taken into consideration, and another option would be watchful waiting rather than immediate surgery. However, approximately one-quarter of lesions diagnosed as DCIS *via* core needle biopsy may be upgraded to IBCs on the final pathology with surgical specimens because the limited number, size, and location of samples may miss IBCs (6, 7). Some patients with a missed diagnosis of IBCs may elect to forgo surgery and pursue watchful waiting, but this management strategy is not safe for these patients (8).

Magnetic resonance imaging (MRI) is a powerful tool for discriminating breast lesions. MRI can noninvasively cover the whole breast with high-spatial-resolution images. Diffusion-weighted imaging (DWI) can provide a surrogate marker for tissue microstructure and cell density by measuring the random movement of water molecules (9). A previous study showed that the apparent diffusion coefficient (ADC) obtained with DWI could be used as a valuable noninvasive quantitative biomarker to assess breast cancer invasiveness (10). However, it is not easy for radiologists to select a representative region of interest (ROI) of a lesion, particularly for nonmass lesions. Differences in ROIs may lead to ADCs that do not truly reflect the lesion microstructure and cell density. Furthermore, tumors interact with the tumor microenvironment, and peritumoral tissue has been indicated to provide helpful information for the diagnosis and prognosis of tumors (11–13), while the conventional method of ADC measurement usually ignores the additional peritumoral information that helps assess invasion.

Deep learning algorithms have displayed excellent performance in image recognition tasks (14). Many convolutional neural network (CNN) models with superior performance exist in deep learning, such as ResNet, AlexNet, VGG, and InceptionV3. CNNs can scan all the pixels of the images using convolution kernels and perceive the global information of the images. Thus, CNNs may offer a promising alternative to discriminate between DCIS and IBCs because of their advantages of being efficient, accurate, and reproducible. Accordingly, this study aimed to determine whether CNN applied to breast DWI can aid in the preoperative differentiation of DCIS and IBCs.

## Materials and Methods

### Patients

**Figure 1:**
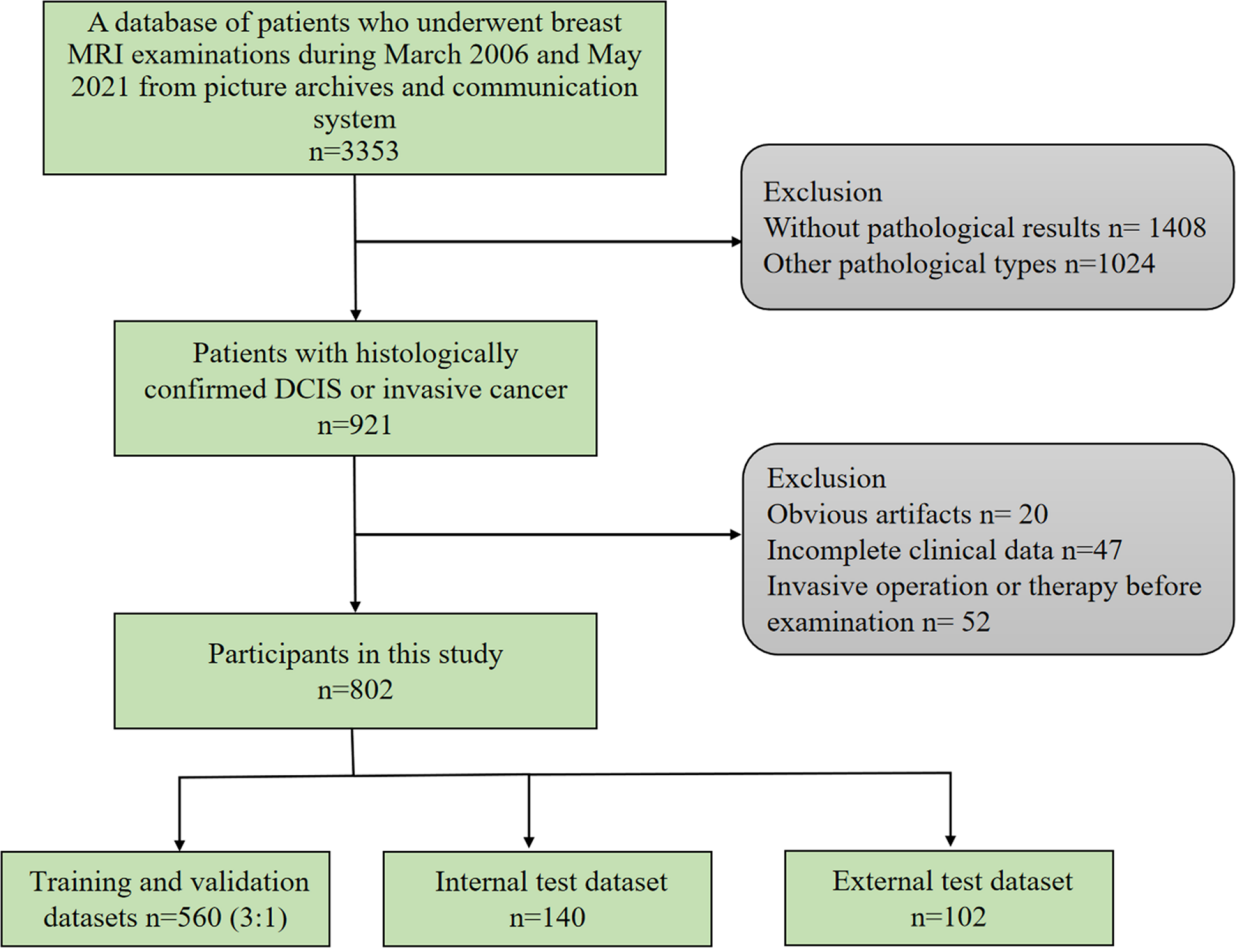
Flowchart of inclusion and exclusion.

This study was approved by the ethics committee of our hospital. The requirement for obtaining informed consent from patients was waived. We retrospectively searched for breast MRI examinations using the picture archiving and communication system. The inclusion criteria for this study were as follows: 1) histologically confirmed pure DCIS or pure IBC; 2) preoperative dynamic contrast-enhanced MRI examination. The exclusion criteria were as follows: 1) preoperative endocrine therapy, chemotherapy, or radiotherapy; 2) preoperative invasive breast operation; 3) incomplete clinical data; 4) obvious artifacts in MR images (**Figure 1**). From March 2006 to June 2019, 700 lesions from 700 patients with primary breast cancers were included, of which 400 lesions were IBCs and 300 lesions were DCIS. We randomly selected 560 lesions as the training and validation sets (ratio of 3 to 1) and 140 lesions as the internal test set. From July 2019 to May 2021, an independent external test cohort of 102 patients with primary breast cancers from our hospital was selected as the primary cohort with the same criteria. A total of 102 lesions from these patients were included in this study.

### MR Image Acquisition

Breast MRI examinations of the primary cohort were performed using 3.0 T superconducting MR scanners (Verio or Trio; Siemens Medical Systems, Erlangen, Germany) with a dedicated breast surface coil (4-channel or 4-channel coils). All the breast MRI examinations of the external test cohort were performed using 3.0 T superconducting MR scanners (Prisma; Siemens Medical Systems, Erlangen, Germany) with a dedicated breast surface coil (18-channel coils). All the patients were scanned in the prone position. After the standard bilateral T2-weighted (T2W) axial and DWI fat-saturated axial sequences with T1-weighted (T1W) gradient-echo VIEWS sequences, a dynamic protocol was performed with six dynamic acquisitions, one before and five immediately after an elbow vein bolus injection of gadolinium-dimeglumine (GE Healthcare) equal to 0.1 mmol per kg body weight, followed by a 20 ml saline flush. The scanning parameters of DWI are summarized in **Table 1**.

**Table 1:** Scanning parameters of diffusion-weighted imaging protocols on 3.0 Tesla scanners.

	Trio	Verio	Prisma
Orientation	Axial	Axial	Axial
Repetition time (msec)	5000	4300	6400
Echo time (msec)	66	80	60
Field of view (cm)	34 × 34	34 × 34	34 × 34
Matrix size	256 × 256	256 × 256	256 × 256
Echo train length	1	1	1
Slice thickness (mm)	4.0	5.0	4.0
b value (s/mm^2^)	0, 1000	0, 1000	0, 1000
Gap (mm)	1.0	1.0	1.5

### Definition of ROIs and Mean ADC

**Figure 2:**
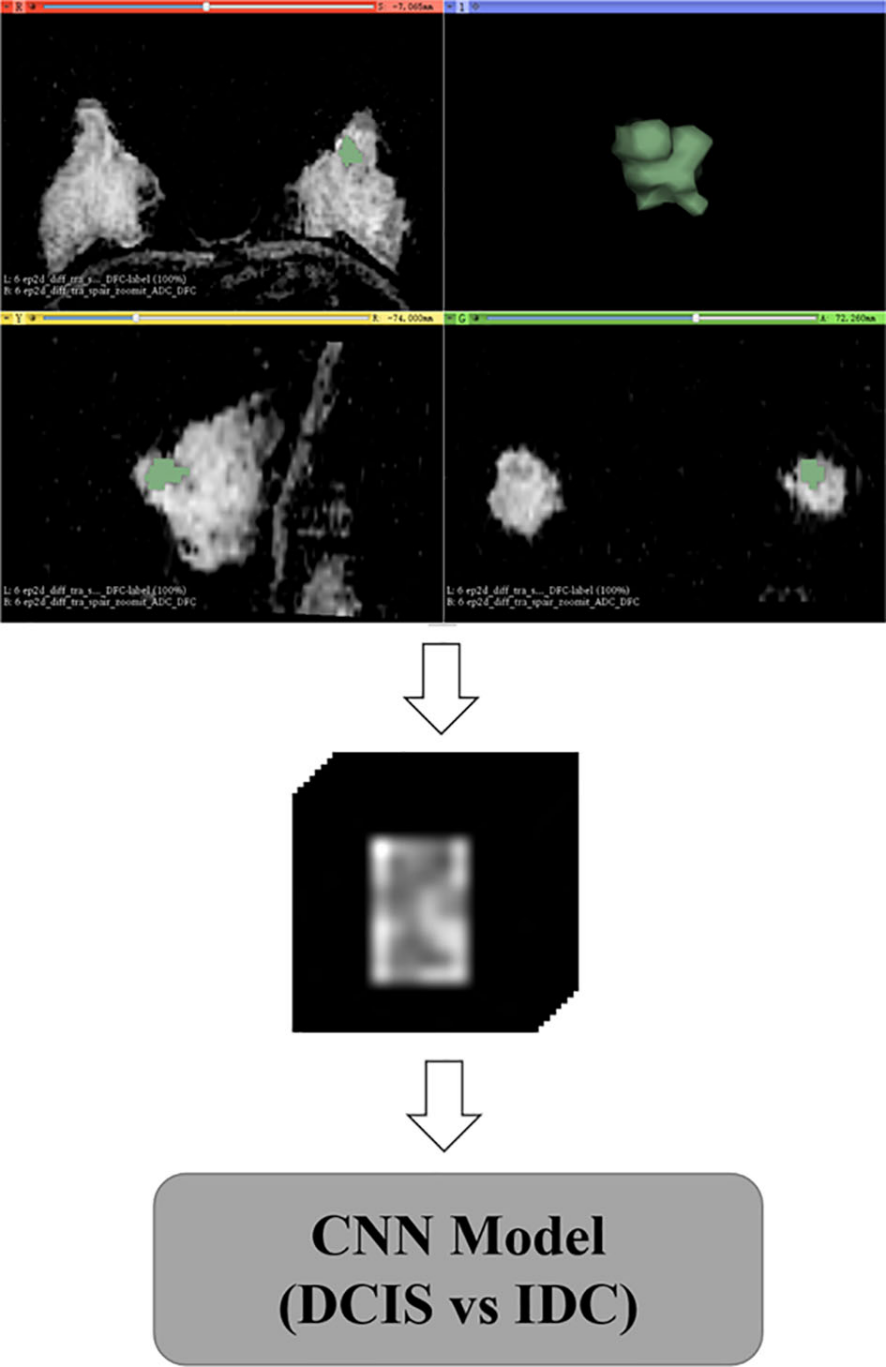
Delineation and preprocessing of regions of interest on apparent diffusion coefficient images.

For the delineation and confirmation of ROIs of the lesions on ADC images, the images of T1W, T2W, and dynamic contrast-enhanced sequences were referred. Open-source software (3D Slicer; https://www.slicer.org/) was used to draw polygon ROIs for the CNN model. The polygon ROIs were drawn to cover whole lesion slice-by-slice by a radiologist with five years of experience in breast MRI analysis blinded to information about histopathology. An example of polygon ROIs is shown in **Figure 2**. Next, the polygon ROIs were confirmed by a radiologist with more than 15 years of experience in breast MRI analysis. To measure the mean ADCs of the breast lesions, round ROIs with sizes ranging from 16 to 225 mm^2^ were manually placed slice-by-slice for the whole lesion volume by the radiologist with over 15 years of experience, while cystic, necrotic, fatty, and hemorrhagic areas were avoided. The ADCs were measured directly from the picture archiving and communication system of the hospital. The mean ADCs were defined as the sum of the ADCs of all ROIs divided by the number of ROIs.

### Data Preprocessing

Data augmentation was applied to the training and validation sets during the training, with random rotation from -10 to 10 degrees, stretching from 0.8 to 1.2, and shifting from -10 to 10 pixels. After the geometric image transformations, the original size of the training and validation sets was expanded five times. The data augmentation strategy can help prevent network overfitting and avoid interference from various sources of noise to improve the robustness of the model (15, 16). Based on polygon ROIs of each lesion, a block centered at the center of the lesion containing the whole lesion region was cropped from MR images, and all blocks were reshaped to a size of 128×128×22 by zero-padding (**Figure 2**).

### Network Architecture

**Figure 3:**
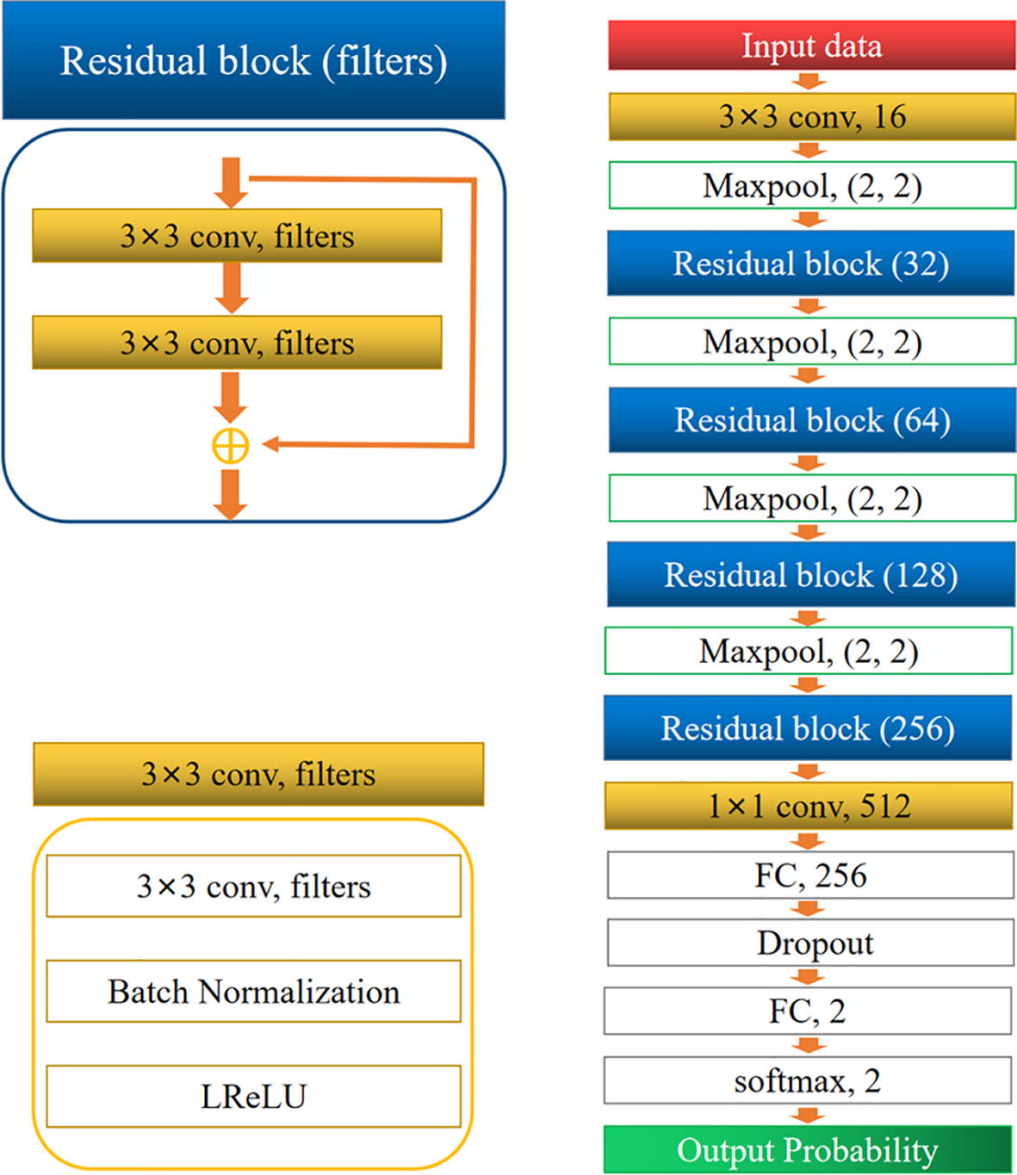
Architecture of the convolutional neural network.

The architecture of the network is shown in **Figure 3**. It comprised two convolution layers, four residual blocks, four max-pooling layers, two fully connected layers, and one softmax layer. Dropout was performed for the first fully connected layers to avoid overfitting. Finally, the softmax layer was used to obtain the probability of classification. The residual block was inspired by ResNet (17). All convolution layers were followed by a batch normalization (BN) layer (18), and a leaky rectified linear unit (LReLU) was used as the activation function.

### Model Training and Testing

All preprocessing was conducted in Python (version 3.7.0; Python Software Foundation, Wilmington, Del) using PyTorch (version 1.4.0). The blocks from the training sets were fed into the network to adjust the weight of the network. Before feeding into the network, all the blocks were standardized by subtracting the mean and dividing by the standard deviation. During the training process, the ADAM algorithm with a learning rate of 0.001 was used to minimize the loss (cross-entropy) function, with a mini-batch size of 32. Finally, the model with the lowest validation loss was selected. During the training phase, an L2 regularization strategy on weight and bias was applied to prevent overfitting. The blocks from the two test sets were fed into the network to output the predicted probability of every class, and the class with the highest probability was chosen as the classification result. All the experiments were performed using a workstation equipped with two NVIDIA TITAN XP GPUs.

### Statistical Analysis

All statistical analyses were performed using SPSS (IBM SPSS Statistics for Windows, v.25.0, Armonk, NY) and Python. We compared the diagnostic performance of the CNN model and mean ADC on the internal test set and external test set. The gold standard for the diagnosis of breast lesions was the postoperative histopathology result, and the classification results derived from the CNN models and the mean ADC were compared with the postoperative histopathology results. The area under the curve (AUC) and its 95% confidence interval (CI), sensitivity, specificity, positive predictive value (PPV), negative predictive value (NPV), F1 score, kappa value, and accuracy were calculated. The cutoff value was determined by maximizing Youden’s index. Significant differences between AUCs were compared by DeLong’s test (19). We analyzed the clinical characteristics of patients with primary breast cancers. Welch’s t test or Student’s t test was used for continuous variables, and Pearson’s chi-squared test was used for categorical variables. A p value < 0.05 was considered statistically significant.

## Results

### Clinicopathologic Data

The mean age was 48.5 years (range, 29–84 years) for patients in the training and validation sets, 50.4 years (range, 31–79 years) for patients in the internal test set, and 50.7 years (range, 35–74 years) for patients in the external test set. Among all 802 patients, 448 (55.8%) had undergone lumpectomy, and 354 (44.2%) had undergone mastectomy. Surgical specimens revealed 253 (31.5%) invasive lobular cancers, 202 (25.2%) invasive ductal cancers, and 347 (43.3%) DCIS cases. Among all the lesions, 688 (85.8%) presented as mass lesions, whereas 114 (14.2%) were nonmass lesions. The clinicopathological characteristics of all participants are listed in **Table 2**.

**Table 2:** Clinicopathological characteristics of the participants.

Characteristic	Tra and Val Sets	Internal Test Set	External Test Set	*P* value
Patients	560	140	102	
Age	48.5 (29–84)	50.4 (31–79)	50.7 (35–74)	0.321
<40 y	118 (21.1)	26 (18.6)	13 (12.7)	
40-49 y	193 (34.5)	47 (33.6)	38 (37.3)	
50-59 y	142 (25.3)	44 (31.4)	34 (33.3)	
≥60	107 (19.1)	23 (16.4)	17 (16.7)	
Menopausal status				0.572
Premenopausal	293 (52.3)	67 (47.9)	55 (53.9)	
Postmenopausal	267 (47.7)	73 (52.1)	47 (46.1)	
Tumor size				0.848
≤2.0 cm	258 (46.1)	61 (43.6)	41 (40.2)	
2.1-4.0 cm	253 (45.2)	67 (47.9)	51 (50.0)	
>4.0 cm	49 (8.7)	12 (8.5)	10 (9.8)	
Lesion position				0.053
Right	296 (52.8)	75 (53.5)	41 (39.9)	
Left	264 (47.2)	65 (46.5)	61 (60.1)	
Morphology				0.683
Mass	484 (86.5)	119 (85.3)	85 (83.1)	
Non-mass	76 (13.5)	21 (14.7)	17 (16.9)	
Histologic type				0.619
Invasive	316 (56.4)	84 (60.0)	55 (53.9)	
DCIS	244 (43.6)	56 (40.0)	47 (46.1)	
Tumor grade				0.063
Low	87 (15.5)	28 (19.9)	23 (23.1)	
Moderate	298 (53.3)	81 (57.8)	45 (43.8)	
High	175 (31.2)	31 (22.3)	34 (33.1)	

Tra and val sets, Training and validation sets; DCIS, ductal carcinoma in situ.

### CNN Model and Mean ADC

**Figure 4:**
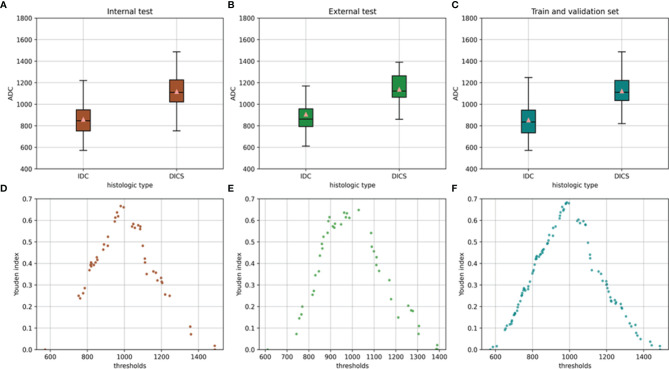
Mean ADC of the invasive breast cancer group and breast ductal carcinoma *in situ* group in the internal test set **(A)** external test set **(B)** and training and validation sets **(C)**. Relationship between the Youden index and threshold in the internal test set **(D)** external test set **(E)** and training and validation sets **(F)**.

**Figure 5:**
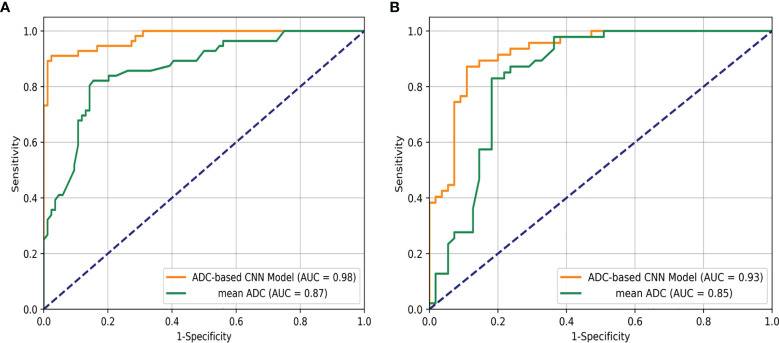
Receiver operating characteristic curve analysis for the differentiation of breast ductal carcinoma *in situ* and invasive breast cancers in the internal test set **(A)** and the external test set **(B)**.

An overview of the performance of the CNN model and mean ADC is shown in **Table 3**. Regarding the differentiation of IBC and DCIS in 140 patients in the internal test set, the CNN model yielded excellent performance, with an AUC of 0.977 (95% CI: 0.957, 0.998), a sensitivity of 0.893, a specificity of 0.929, a PPV of 0.949, an NPV of 0.852, an F1 score of 0.908, a kappa value of 0.809 and an accuracy of 0.907. In the internal test set, the mean ADC of the IBC group was 0.859×10^−3^ mm^2^/s (standard deviation, 0.148×10^−3^ mm^2^/s); in the DCIS group, it was 1.118×10^−3^ mm^2^/s (standard deviation, 0.169×10^−3^ mm^2^/s) (**Figure 4A**). IBC showed significantly lower ADCs than DCIS (P < 0.001). The optimal threshold for an ADC of 0.980×10^−3^ mm^2^/s was applied to the internal test set (**Figure 4D**), and the mean ADC at this threshold showed an AUC of 0.866 (95% CI: 0.805, 0.927), a sensitivity of 0.845, a specificity of 0.821, a PPV of 0.877, an NPV of 0.780, an F1 score of 0.836, a kappa value of 0.661 and an accuracy of 0.836. As shown in **Figure 5A**, the performance of the CNN model was significantly better than that of the mean ADC (P = 0.001).

**Table 3:** Performance of the CNN model and mean ADC.

	Internal Test Set (140)		External Test Set (102)		Tra and Val Sets (560)
	CNN Model	Mean ADC	CNN Model	Mean ADC	Mean ADC
Accuracy	0.907	0.836	0.902	0.824	0.845
Sensitivity	0.893	0.845	0.873	0.818	0.864
Specificity	0.929	0.821	0.894	0.829	0.820
PPV	0.949	0.877	0.906	0.849	0.861
NPV	0.852	0.780	0.857	0.796	0.823
F1 score	0.908	0.836	0.882	0.824	0.845
kappa value	0.809	0.661	0.764	0.646	0.684
AUC (95% CI)	0.977 (0.957-0.998)	0.866 (0.805-0.927)	0.926 (0.876-0.976)	0.845 (0.766-0.925)	0.868 (0.838-0.899)

AUC, area under the receiver operating characteristic curve; CI, confidence interval; CNN, convolutional neural network; NPV, negative predictive value; PPV, positive predictive value; Tra and val sets, training and validation sets; ADC, apparent diffusion coefficient.

Regarding the identification of IBC and DCIS in 102 patients in the external test set, the CNN model also achieved good performance, with an AUC of 0.926 (95% CI: 0.876, 0.976), a sensitivity of 0.873, a specificity of 0.894, a PPV of 0.906, an NPV of 0.857, an F1 score of 0.882, a kappa value of 0.764 and an accuracy of 0.902. The mean ADC was also significantly lower in the IBC group than in the DCIS group in the external test set (P < 0.001). The mean ADC in the IBC group was 0.907×10^−3^ mm^2^/s (standard deviation, 0.178×10^−3^ mm^2^/s), while the mean ADC in the DCIS group was 1.138×10^−3^ mm^2^/s (standard deviation, 0.139×10^−3^ mm^2^/s) (**Figure 4B**). The optimal threshold for an ADC of 1.029 × 10^−3^ mm^2^/s was applied to the external test set (**Figure 4E**), and the mean ADC at this threshold showed an AUC of 0.845 (95% CI: 0.766, 0.925), a sensitivity of 0.818, a specificity of 0.829, a PPV of 0.849, an NPV of 0.796, an F1 score of 0.824, a kappa value of 0.646 and an accuracy of 0.824. As shown in **Figure 5B**, the performance of the CNN model was slightly better than that of the mean ADC, while there was no significant difference between them (P = 0.096).

Regarding the identification of IBC and DCIS in 560 patients in the training and validation sets, the mean ADC in the IBC group was 0.853×10^−3^ mm^2^/s (standard deviation, 0.159×10^−3^ mm^2^/s). In the DCIS group, the mean ADC was 1.123×10^−3^ mm^2^/s (standard deviation, 0.169×10^−3^ mm^2^/s) (**Figure 4C**). IBC showed significantly lower ADCs than DCIS (P < 0.001). The optimal threshold for an ADC of 0.985×10^−3^ mm^2^/s was applied to the training and validation sets (**Figure 4F**), and the mean ADC at this threshold showed an AUC of 0.868 (95% CI: 0.838, 0.899), a sensitivity of 0.864, a specificity of 0.820, a PPV of 0.861, an NPV of 0.823, an F1 score of 0.845, a kappa value of 0.684 and an accuracy of 0.845.

**Figure 6:**
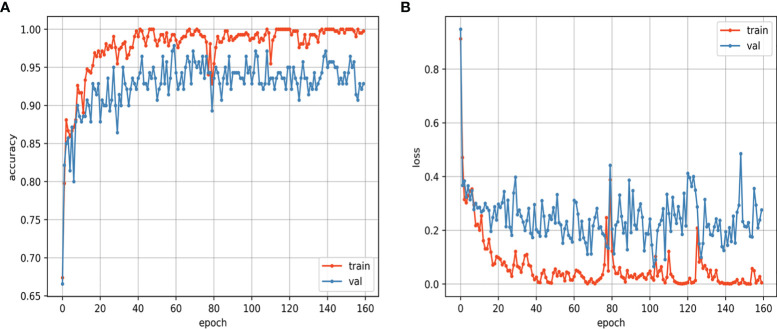
Loss curves **(A)** and accuracy curves **(B)** of the training and validation sets.

The training and validation curves of the CNN model that reflect the process of training are shown in **Figure 6**. As the training epoch continued, the accuracy curves of the training and validation sets gradually became stable after the rapid rise and slow rise, and the loss curves of the training and validation sets gradually became stable after the rapid decline and slow decline. The similar trends of the two loss curves suggest that the CNN model was not overfitted. The training was performed for 160 epochs, and the CNN model learned the entire training set once at each epoch. The CNN model achieved the best accuracy at the 60th epoch, and the assessments on the internal test set and external test set were based on this best model. For the CNN model, the training of the model took approximately 48 hours, and the prediction took approximately one second for a single lesion. For the mean ADC, it took approximately 3 to 8 minutes to perform the measurement and calculation for a single lesion.

## Discussion

This study showed the exciting utility of the CNN model in identifying IBC and DCIS. The CNN model showed good performance, with AUCs of 0.977 and 0.926, sensitivities of 0.893 and 0.873, and specificities of 0.929 and 0.894 for the internal test set and the external test set, respectively. The mean ADCs of DCIS were significantly higher than those of IBC in our study. This finding is in concurrence with those of previous studies (20). The mean ADC of the internal test set and external test set showed AUCs of 0.866 and 0.845, sensitivities of 0.845 and 0.818, and specificities of 0.821 and 0.829, respectively. Overall, the performance of the CNN model was better than that of the mean ADC. Our study successfully developed a model for discriminating IBC and DCIS in patients with breast cancer using CNN, and our results showed an improved performance in the assessment of pathological subtypes of breast cancer based on ADC images from the preoperative scans of the patients.

DWI is a quantitative measurement technique that depicts the Brownian motion of water molecules, and the ADC indirectly shows the integrity of cell membranes and degree of cell crowding (21). Therefore, the ADC provides some insight into the biological characteristics of breast lesions. Although the mean ADC has helped considerably to differentiate the pathological subtypes of breast cancer, it represents only the average measurement of voxels in the ROI area and does not consider the spatial relationship among voxels. The CNN model obtained a significantly higher AUC than the mean ADC in both the internal test set and external test set, indicating that much spatial information hidden in the ADC images of patients with primary breast cancer is useful to differentiate among pathological subtypes. This finding was also observed in a previous study (22).

ADC is an objectively and quantitatively measured variable that is less dependent on reader and interobserver variabilities than conventional morphologic features, such as shape, margin, or distribution pattern (10, 23). However, the variability of the ADC in breast DWI due to the signal-to-noise ratio, motion, off-isocenter effects, different field strengths, sequence variants of the different platforms, and inconsistencies in the ROI definition cannot be ignored (24, 25). In our study, for both the internal test set and external test set, the mean ADC achieved satisfactory performance, but the mean ADC and optimal thresholds were quite different. Our findings were consistent with those of some previous studies, which showed significant differences in the ADCs of lesions between IBC (ranging from 0.65 to 1.31×10^−3^ mm^2^/s) and DCIS (ranging from 0.83 to 1.59 × 10^−3^ mm^2^/s) (20, 26, 27). These substantial heterogeneities indicate that standardized measurement protocols, centralized quality control and centralized analyses are needed for different medical institutions, and different thresholds will be needed for ADC images of patients with primary breast cancer obtained from different scanners, protocols, and field strengths (28, 29).

In our study, the CNN model eliminated the challenge of artificially selecting the optimal ADC cutoff value and had similar performance on the internal test set and external test set. ADC images were normalized to the range from 0 to 1. The normalization method can partially eliminate the difference in data obtained from different scanners. Additionally, unlike the MRI signal of T1W and T2W sequences, which is nonlinearly related to proton density, relaxation time, time of repetition, and time of echo, ADC is an inherent physical value (22). Each ADC of a pixel-by-pixel volume has the same drift tendency when using different scanners, protocols, and field strengths. The advantage of the CNN model is that it considers the spatial relationship of a pixel-by-pixel volume in the task of identification and, may further ignore the differences from the grayscale drift of ADC images. Therefore, ADC images may be less affected by different scanners and could be good candidates to construct CNN models using data from multiple sources.

Although the manual placement of round ROIs slice-by-slice is a common method of measurement, the definition of these ROIs is very tedious and time-consuming. Additionally, operator variability in the definition of these ROIs is a significant factor currently limiting the reproducibility of ADC measurements. In our study, the blocks were generated based on polygon ROIs for the CNN model, and these blocks contained some peritumoral parenchyma. This method not only ensures a certain degree of repeatability but also obtains additional peritumoral information that helps predict invasion. The peritumoral/tumor ADC ratio is likely related to the extensive hyaluronan accumulation and biological aggressiveness of breast cancer (30). The peritumoral environment contain critical and rich information related to tumor invasiveness, including lymphovascular invasion, angiogenesis, lipids, and inflammatory components, which can be used for diagnosis or prediction (13, 31). Previous studies have confirmed that combining intratumoral and peritumoral regions can achieve significantly better performance in different tasks (32, 33).

This study has several limitations. First, this study had a retrospective design, and our results were based on a limited number of patients. Therefore, larger sample size studies are needed in the future to confirm the results. Second, selection bias may be present in our study, because these patients were not consecutive cases. Third, although the possible benefits of additional information from the peritumoral regions were considered, the blocks were not the best choice for sampling peritumoral information. An automatic segmentation algorithm based on certain standards is a promising solution, but the accuracy and stability of these algorithms still need improvement.

## Conclusion

In summary, the ADC-based CNN model can improve the differentiation of IBC from DCIS with higher accuracy and less time. This strategy seems to be an effective alternative, valuable, noninvasive method to assess breast cancer invasiveness. Thus, our ADC-based CNN model has great potential to reduce overdiagnosis and is a potentially useful decision support tool in clinical applications.

## Data Availability Statement

The data analyzed in this study is subject to the following licenses/restrictions: The datasets presented in this article are not readily available because of the privacy of patient information. Requests to access these datasets should be directed to corresponding author GL.

## Ethics Statement

The studies involving human participants were reviewed and approved by The Institutional Review Board of Huadong Hospital ffiliated with Fudan University. Written informed consent for participation was not required for this study in accordance with the national legislation and the institutional requirements.

## Author Contributions

GL and TC: study conception and design. HY and WH: data collection and analysis. HY and ZX: image processing and modeling. HY: manuscript writing. YJ: statistical analysis. All authors contributed to the article and approved the submitted version.

## Funding

This work was financially supported by National Natural Science Foundation of China (81771816).

## Conflict of Interest

The authors declare that the research was conducted in the absence of any commercial or financial relationships that could be construed as a potential conflict of interest.

## Publisher’s Note

All claims expressed in this article are solely those of the authors and do not necessarily represent those of their affiliated organizations, or those of the publisher, the editors and the reviewers. Any product that may be evaluated in this article, or claim that may be made by its manufacturer, is not guaranteed or endorsed by the publisher.
